# Supplementary material for: Identification and functional analysis of non-coding regulatory small RNA FenSr3 in Bacillus amyloliquefaciens LPB-18
Source: PeerJ. 2023 May 15;11:e15236. doi: 10.7717/peerj.15236 (PMC10194069; doi:10.7717/peerj.15236)
Supplement: Supplemental Information 4 [file peerj-11-15236-s004.zip › KO/CK-vs-T1_map/map02024.html]

KEGG PATHWAY: Quorum sensing - Reference pathway


|  |  |
| --- | --- |
| **Quorum sensing - Reference pathway** |  |

[
Pathway menu
| Organism menu
| Pathway entry
| Show description
| User data mapping
]

Quorum sensing (QS) is a regulatory system that allows bacteria to share information about cell density and adjust gene expression accordingly. All QS bacteria produce and release chemical signal molecules called autoinducers (AIs) that increase in concentration as a function of cell density. The most commonly studied AIs belong to one of the following three categories: acylated homoserine lactones, also referred to as AI-1, used by Gram-negative bacteria; peptide signals, used by Gram-positive bacteria; and AI-2, used by both Gram-negative and Gram-positive bacteria. QS can be divided into at least 4 steps: production of AIs by the bacterial cell; release of AIs, either actively or passively, into the surrounding environment; recognition of AIs by specific receptors; and leading to changes in gene regulation once they exceed a threshold concentration. The processes controlled by QS include virulence, competence, conjugation, antibiotic production, motility, sporulation, and biofilm formation.

|  |  |  |
| --- | --- | --- |
| Reference pathway | 184% 150% 122% 100% 82% 67% 55% | 图片下载 |
